# Metabolomics Reveals the Efficacy of Caspase Inhibition for Saikosaponin D-Induced Hepatotoxicity

**DOI:** 10.3389/fphar.2018.00732

**Published:** 2018-07-06

**Authors:** Qian-qian Zhang, Wan-qiu Huang, Yi-qiao Gao, Zhao-di Han, Wei Zhang, Zun-jian Zhang, Feng-guo Xu

**Affiliations:** ^1^Key Laboratory of Drug Quality Control and Pharmacovigilance (Ministry of Education), China Pharmaceutical University, Nanjing, China; ^2^State Key Laboratory of Natural Medicine, China Pharmaceutical University, Nanjing, China; ^3^State Key Laboratory for Quality Research in Chinese Medicines, Macau University of Science and Technology, Macau, Macau

**Keywords:** saikosaponin d, hepatotoxicity, caspase inhibition, metabolomics, bile acids, inflammation

## Abstract

Saikosaponin d (SSd) is a major hepatoprotective component of saikosaponins derived from *Radix Bupleuri*, which was also linked to hepatotoxicity. Previous studies have demonstrated that caspases play a key role in SSd-induced liver cell death. Our *in vitro* and *in vivo* studies also showed that treatment with caspase inhibitor z-VAD-fmk could significantly reduce the L02 hepatocyte cells death and lessen the degree of liver damage in mice caused by SSd. In order to further reveal the underlying mechanisms of caspase inhibition in SSd-induced hepatotoxicity, mass spectrometry based untargeted metabolomics was conducted. Significant alterations in metabolic profiling were observed in SSd-treated group, which could be restored by caspase inhibition. Bile acids and phospholipids were screened out to be most significant by spearman correlation analysis, heatmap analysis and S-Plot analysis. These findings were further confirmed by absolute quantitation of bile acids via targeted metabolomics approach. Furthermore, cytokine profiles were analyzed to identify potential associations between inflammation and metabolites. The study could provide deeper insight into the hepatotoxicity of SSd and the efficacy of caspase inhibition.

## Introduction

*Radix Bupleuri*, one of the famous crude drugs in the prescriptions of traditional Chinese medicines, has been used in China for over 2000 years (Yu et al., 2017). However, it has been increasingly reported liver injury in clinical context along with wide usage of *R. Bupleuri* for the treatment of liver disease (Itoh et al., 1995; Lee et al., 2011). SSd is a typical toxic saikosaponin derived from *R. Bupleuri* that could cause liver injury (Chen et al., 2013; Zhang F. et al., 2016; Yuan et al., 2017). Chen et al. have proved that SSd could interrupt PDGF-βR signaling leading to mitochondrial apoptosis and activated Fas, caspase-8, and Bid, contributing to cytochrome c release and caspase-3 activation in hepatocytes. Zhang et al. and Li et al. also found that SSd could cause liver injury and hepatocyte death in animal experiments.

More and more evidences have demonstrated that caspases play a key role in pathological cell death caused by apoptosis, necroptosis or programmed necrosis in diseased liver (Kopec et al., 2017; Woolbright et al., 2017). Moreover, inhibition of caspases has routinely been tested in multiple models of liver injuries as a potential means to lessen the degree of liver damage (Cheng et al., 2009; Witek et al., 2009; Barreyro et al., 2015). Our preliminary studies have showed that caspase-1 contributed to the hepatocyte cells death induced by SSd, which could be effectively alleviated by the caspase inhibitor z-VAD-fmk. Z-VAD-fmk is a pan caspase inhibitor that could serve as caspase inhibiton and was also reported against caspase-1 activation related injury, such as inflammation (Kim et al., 2016). However, the way as to how can caspase inhibition reduce SSd-induced hepatotoxicity remain still unclear.

*In vitro* experiment reflected the toxicity of SSd and efficacy of caspase inhibition to hepatocytes quickly and intuitively, but it cannot comprehensively and objectively reflect the metabolic regulation mechanism *in vivo*. Metabolomics aims to comprehensively monitor alterations at the metabolic levels in response to endogenous or exogenous stimuli and link metabolic disruptions to biological mechanisms (Nicholson et al., 2002; Patti et al., 2012). Therefore, metabolomics might offer a new and comprehensive insight into the hepatotoxicity of SSd and efficacy of caspase inhibition from the aspect of metabolic regulation (Dunn et al., 2012; Creek, 2013).

Hence, in the present study, integrated gas and liquid chromatography mass spectrometry-based metabolomics approach was used to identify the metabolic profiling alterations that are in close relationship SSd-induced hepatotoxicity. The potential associations between inflammation and metabolites were also further analyzed. Based on our results, bile acids and lysophosphatidylcholines (LPCs) were the most significantly in caspase inhibition reduced inflammation-related liver injury, which caused by SSd.

## Materials and Methods

### Chemicals and Reagents

Saikosaponin d (purity > 98%) was purchased from Sichuan Victory Biological Technology Co., Ltd. (Chengdu, China). Z-VAD-fmk was obtained from APExBIO (Houston, United States), and all other chemicals were obtained from Sigma-Aldrich (St. Louis, MO, United States). Methanol, acetonitrile (HPLC grade) were purchased from Merck (Germany). Distilled water was filtered through a Milli-Q system from EMD Millipore Corporation (Billerica, MA, United States).

### Animal Experiments and Sample Collection

All animal experiments were performed in accordance with the institutional guidelines for the care and use of laboratory animals. And all experimental protocols were approved by the Animal Ethics Committee of China Pharmaceutical University and carried out under the Guidelines for Care and Use of Laboratory Animals. A total of 21 male ICR mice (18–22 g body weight) were obtained from Nanjing Qinglong lab. Animal Co., Ltd. (Jiangsu, China). All animals were maintained under specific-pathogen-free conditions and were given a standard diet and tap water *ad libitum* for 1 week before experiments. After 1 week adaptive feeding, they were randomly divided into three groups (*n* = 7).

Mice in SSd-treated group (group M) and caspase inhibition group (group MF) were received a single intraperitoneal injection of 25 mg/kg of SSd at time 28 h. The caspase inhibition group was given z-VAD-fmk as a series of two intraperitoneal injections of 10 mg/kg at 0 h and 24 h, the control group (group C) was given the blank solvent. Body weight was monitored every day before intraperitoneal injection. At the time of 52 h, mice were sacrificed. On collection, blood samples were collected and serum was separated by centrifugation (8000 g × 10 min, 4°C). A small portion of the liver was removed for histopathological analysis by fixation with 10% formalin and the remaining liver was cut in pieces and rapidly frozen with liquid nitrogen for extraction of hepatic proteins. The scheme of the whole experiment was shown in Supplementary Figure S1.

### Histopathological Examination and Biochemical Analysis

Livers for histopathologic examination were fixed and preserved in 10% neutral buffered formalin, processed and trimmed, embedded in paraffin, sectioned to a thickness of approximately 5 mm, and stained with hematoxylin and eosin (HE).

Biochemical analysis was performed on all animals. The enzymatic activities of serum ALT, AST were measured by assay kits (Beyotime Institute of Biotechnology, Shanghai, China).

### Measurement of Caspase-1 Activity

The caspase-1 activity was determined by a caspase-1 assay kit (Beyotime Institute of Biotechnology, Shanghai, China). A piece of liver (10 ± 0.1 mg) was homogenized with 100 μL ice-cold lysis buffer and then was incubated for 5 min. After centrifuged at 20000 g, 4°C for 10 min, the supernatants were retrieved and aliquots corresponding to protein at the concentration of 1-3 mg/mL. 40 μL caspase-1 buffer and 10 μL YVAD-pNA (2 mM) substrate were added to each sample. The samples were then incubated at 37°C for 2 h. The absorbance was recorded at 405 nm using Tecan Infinite 200 Pro (Tecan Group Ltd., Mannedorf, Switzerland). The activity of caspase substrates was expressed as a percentage of enzyme activity compared with the controls.

### Metabolomics Analysis

Analytical methods and instruments for metabolomics analysis were based on our previous studies (Dai et al., 2016; Zhang P. et al., 2016). The key steps include sample preparation, GC-MS, and LC-MS analysis, data preprocessing and analysis, metabolites identification, as shown in Supplementary Information. After metabolic information collection and data preprocessing, the PCA and OPLS-DA models were firstly constructed between control and SSd-treated groups in order to get differential metabolites that related to SSd administration. To explore the metabolic profiles reversed by caspase inhibition, PCA and OPLS-DA models were also built among control, SSd-treated and caspase inhibition groups. An overview of metabolites reversed by z-VAD-fmk was represented in a heatmap.

### Data Quality Evaluation in Metabolomics Analysis

For validating the stability of the analysis process, quality control (QCs) samples were prepared by mixing equal aliquots of all serum samples and were analyzed in the same way as other samples. QCs were injected ten times before the batch process and injected one time every eight samples during the analysis process, to monitor the stability of sample preparation and instrument (Cui et al., 2017). Besides, the common metabolites detected by both LC-MS and GC-MS methods were cross-validated by correlation analysis (Wang et al., 2015).

### Quantitative Evaluation of Toxicity and Efficacy Based on Relative Distance Values

After serum metabolomics analysis, GC-MS data and LC-MS data were integrated as the whole dataset. In order to translate the direct phenotypic graph to quantifiable data, relative distance values (RDV) of different groups in 3D-PCA score plots were calculated and used to quantitatively assess the toxicity of SSd and the efficacy of caspase inhibition. RDV of each sample were calculated using the following equation (Aa et al., 2011).

(1) Di = [(xi−xc)2 + (yi−yc)2 + (zi−zc)2]12

where *D_i_* is RDV relative to control; *x_i_, y_i_*, and *z_i_* are the coordinate values in *x, y*, and *z* axis in 3D-PCA score plots; *x_c_, y_c_*, and *z_c_* are the mean coordinate values in *x, y*, and *z* axis of control.

### Quantification of Bile Acids in Serum

Simultaneous quantitative analysis of bile acid species in serum was based on our previous study (as shown in Supplementary Information) (Wang et al., 2017). Serum sample was analyzed on a ZORBAX Eclipse XDB-C18 (150 mm × 2.1 mm, 3.5 μm) (Agilent Technologies) using LC-MS/MS analysis. MS analysis was performed in a triple quadruple TSQ Quantum mass spectrometer (Thermo Fisher, Palo Alto, CA, United States).

### Multiplex Analysis of Serum and Liver Cytokine Levels

Cytokine (IL-1β, IL-6, IL-10, and TNF-α) levels in serum and liver samples were measured by the Milliplex MAP Mouse Cytokine/Chemokine magnetic bead immunoassay kit (MCTOMAG-70K; EMD Millipore, Billerica, MA, United States). All assay plates were run according to the manufacturer’s protocols. Assay results were analyzed by a Luminex 200 (Luminex, Austin, TX, United States) and reported utilizing Luminex xPONENT^®^ software version 3.1.

### Statistical Analysis

Mann–Whitney *U* test, unpaired Student’s test and correlation analysis were using PASW Statistics 18 (SPSS Inc., Chicago, IL, United States). The heat map was used to visualize the change trend between groups utilizing Multi Experiment Viewer v.4.8.

## Results

### Z-VAD-fmk Decreases Caspase-1 Activity and Alleviates SSd-Induced Liver Injury in Mice

**FIGURE 1:**
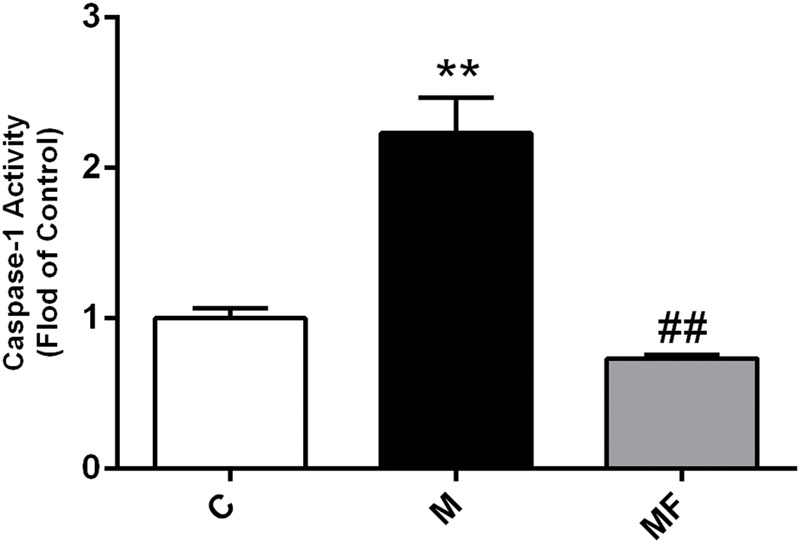
Caspase-1 activity in liver samples among different groups. Values are expressed as the mean ± S.D. Group M vs. C, ^∗^ *P* < 0.05, ^∗∗^*P* < 0.01; Group MF vs. M, ^#^*P* < 0.05, ^##^*P* < 0.01 (*n* = 7 for each group).

Firstly, caspase-1 activity of SSd treatment was increased up to 2.2-fold compared with the control group, which could be completely blocked by z-VAD-fmk (**Figure 1**). Meanwhile, ALT, AST in SSd-treated group were significantly increased compared with those in the control group, accompanied by significantly lower body weight ratio and food intake. And protective effect could be seen in caspase inhibition group with significantly decreased ALT, AST as shown in Supplementary Table S1. Furthermore, HE stained liver sections revealed that several pathological damages were observed after SSd-treatment, including hepatocellular necrosis, inflammatory cell infiltration compared with normal liver of control group (Supplementary Figure S2). While fewer liver damages were observed in caspase inhibition group.

### Data Quality Evaluation in Metabolomics Analysis

The analytical performance of GC-MS and LC-MS was observed to be satisfactory as the QCs were clustered closely in the PCA score plots (Supplementary Figures S3A-C, S4A-C). Meanwhile, the retention time shift was < 0.1 min and the relative standard deviation (RSD) values of all peaks in QCs were < 30%. Methionine and phenylalanine were two metabolites that could be detected by both GC-MS and LC-MS. The correlation analysis showed the results obtained by different analytical approach were in significantly positive correlation. The Pearson/Spearman correlation coefficients for methionine and phenylalanine were 0.777/0.782 and 0.911/0.915, respectively. In collection, the data quality evaluation demonstrated the whole analytical process was stable and reliable.

### Caspase Inhibition Restores Metabolic Changes in SSd-Induced Liver Injury

In order to get global metabolic profile, PCA score plots were firstly applied to display the trends of the samples in control and SSd-treated groups (Supplementary Figures S3A-C). The score plots uncovered that the control and liver damaged mice were obviously separated. The RDV from GC-MS and LC-MS further confirmed that metabolic profiles were markedly altered by SSd exposure and reversed by caspase inhibition (Supplementary Figure S5).

**FIGURE 2:**
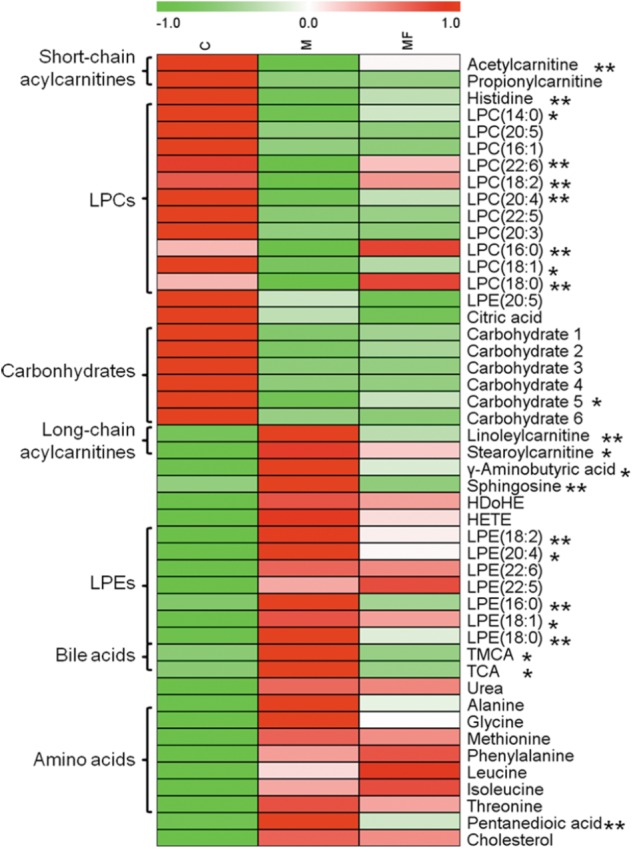
Serum metabolites changes are represented as a heatmap depicting significant metabolite changes induced by SSd (group M vs. C, *p* < 0.05) in C, M and MF groups detected by either LC-MS or GC- MS. Group MF vs. M, ^∗^*p* < 0.05, ^∗∗^*p* < 0.01.

Then, OPLS-DA score plots were applied to identify the metabolites that related to SSd administration (Supplementary Figures S3D-F) and caspase-inhibition (Supplementary Figures S4D-F). Statistical analysis revealed, SSd exposure would lead to the significant change (VIP > 1 and *p* < 0.05) of 47 metabolites (Supplementary Tables S2, S3) and caspase inhibition using z-VAD-fmk could reverse 22 of them (**Figure 2**).

### Bile Acids and LPCs Were Focalized Based on Metabolomics Results

**FIGURE 3:**
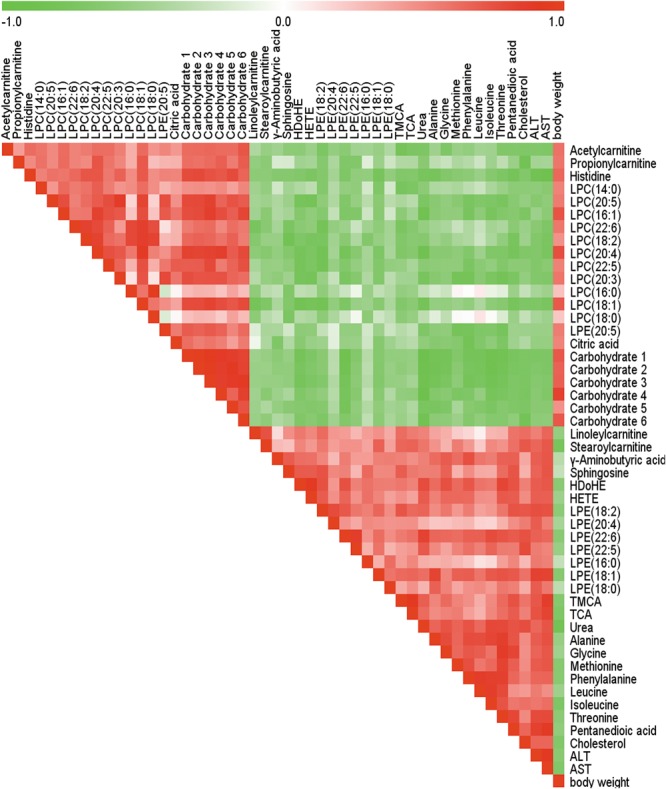
Spearman correlation analysis of serum marker metabolites induced by SSd, ALT, AST as well as body weight. Green squares indicate negative correlations, white squares indicate non-applicable correlations, and red squares indicate positive correlations.

**FIGURE 4:**
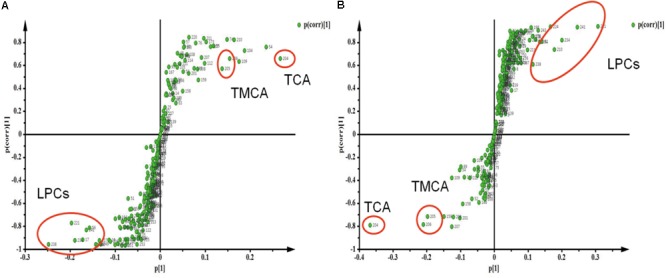
Strategy for identification of interesting metabolites by S-Plot. **(A)** S-Plot of group C and group M. **(B)** S-Plot of group M and group MF. Metabolites are highlighted by circles to demonstrate different regions in the S-plot.

The Spearman correlation analysis was used to identify potential links among altered metabolites, ALT, AST and body weight (**Figure 3**). According to examination index, ALT and AST have a significant positive relationship with bile acids, and was negatively related to LPCs. Meanwhile, S-Plot demonstrated bile acids and LPCs were among the top contributing metabolites that lead to the separation of different groups in OPLS-DA score plot (**Figure 4**). Moreover, as shown in **Figure 4**, bile acids and LPCs could both be significantly reversed by caspase inhibition. All these results indicated that bile acids and LPCs might play essential roles in SSd-induced liver injury.

### Quantification of Bile Acids in Serum

**FIGURE 5:**
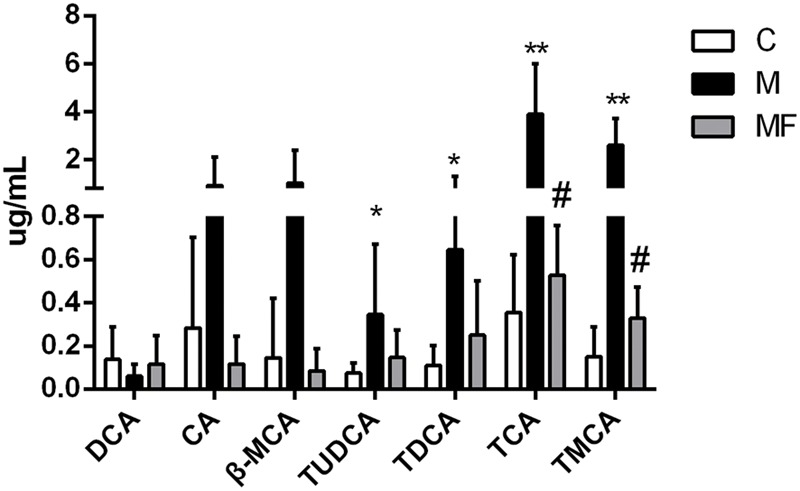
Concentrations of bile acids in serum samples among different groups. Values are expressed as the mean ± S.D. Group M vs. C, ^∗^*P* < 0.05, ^∗∗^*P* < 0.01; Group MF vs. M, ^#^*P* < 0.05, ^##^*P* < 0.01.

Based on untargeted metabolomics data, bile acids including taurocholic acid (TCA), tauromuricholic acid (TMCA) were the top compounds that produced the metabolic differences. We inferred that the change of bile acids might be an important feature in hepatotoxicity of SSd and efficacy of caspase inhibition. In order to uncover the relationship between bile acids profiles and SSd-induced liver damage, absolute quantitation of bile acids was then performed via targeted metabolomics. 7 bile acids were detected and quantified, including deoxycholic acid (DCA), cholic acid (CA), β-Muricholic acid (β-MCA), tauroursodeoxycholic acid (TUDCA), taurodeoxycholic acid (TDCA), TCA, and TMCA. As shown in **Figure 5**, TUDCA, TDCA, TCA, and TMCA were markedly increased as early as 24 h after SSd challenge, thus indicating that elevation of serum bile acids is an early event of liver injury induced by SSd. Meanwhile, TCA, TMCA were significantly reversed by caspase inhibition, which was in consistent with the results of untargeted metabolomics.

### Alternation of Cytokines and Its Associations With Metabolites

**FIGURE 6:**
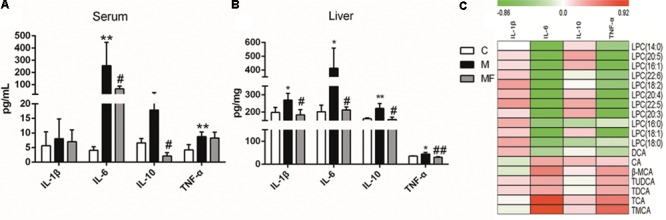
Concentrations of cytokine levels in **(A)** serum and **(B)** liver samples among different groups. **(C)** Spearman correlation analysis between metabolites and cytokines in serum. Values are expressed as the mean ± S.D. Group M vs. C, ^∗^*P* < 0.05, ^∗∗^*P* < 0.01; Group MF vs. M, ^#^*P* < 0.05, ^##^*P* < 0.01.

Caspase-1 is mainly related with activation of IL-1β, an inflammatory cytokine. And the activity of caspase-1 was elevated in SSd treated group, which could be blocked by z-VAD-fmk in caspase inhibition group. Based on the relationship between caspase-1 and inflammation, we focused on the analysis of inflammation in this research. So we tested the serum and liver concentrations of 4 cytokines (IL-1β, IL-6, IL-10, and TNF-α). The data for 4 cytokines in the serum and liver of the three groups was summarized in **Figures 6A,B**. Serum and liver concentrations of IL-6 and TNF-α were significantly elevated, and concentrations of IL-1β and IL-10 in liver were markedly elevated after SSd administration. Caspase inhibition could reverse the change of the cytokines concentrations in different degree. As previous studies have demonstrated that inflammation, bile acids and LPCs are in high correlation in liver injury (Patterson et al., 2011; Matsubara et al., 2012). Thus, spearman correlation analysis was used to identify potential links between metabolites and cytokines in serum (**Figure 6C**). As showed in **Figure 6C**, IL-6 and TNF-α were determined to correlate negatively with LPCs and were also statistically linked positively to bile acids.

## Discussion

**FIGURE 7:**
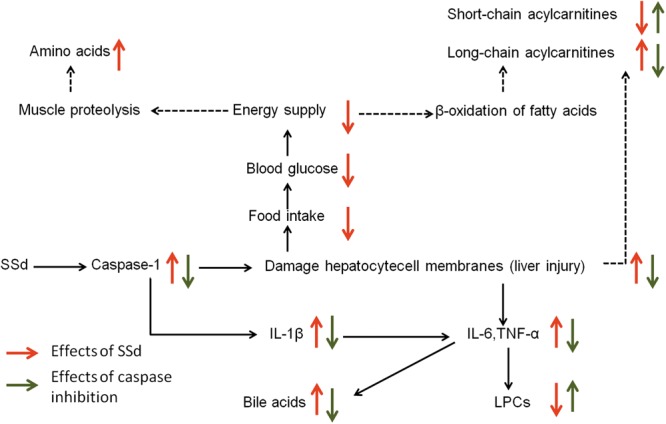
The perturbed metabolic pathways in response to SSd treatment as well as caspase inhibition. Elevation (up arrows) and reduction (down arrows) of the levels of metabolites observed in rats are indicated.

In the current study, biochemical analysis and histopathological examination confirmed that caspase inhibition could ameliorate liver damage caused by SSd. By using a metabolomics approach, 47 altered metabolites including carbohydrates, amino acids, acylcarnitines, bile acids, and various phospholipids were identified after SSd exposure. Meanwhile, 22 metabolites could be significantly reversed by caspase inhibition. Our inferred comprehensive mechanisms were summarized in **Figure 7** based on our investigation.

The decreased carbohydrates and the increased amino acids could be related to the reduced food intake (**Figures 2, 3** and Supplementary Table S1), for the acute liver injury caused by SSd treatment. And elevated levels of amino acids might represent the enhanced muscle proteolysis (Fontana et al., 1996; Zira et al., 2013) as to maintain the energy supply in the condition of reduced carbohydrates intake. However, caspase inhibition could only slightly reverse the altered levels of carbohydrates and amino acids (**Figure 2**). This indicated that caspase inhibition-mediated protective effect in SSd-induced liver damage had slight influence on food intake.

Another important feature in SSd-treated group was decreased short-chain acylcarnitines (acetylcarnitine, propionylcarnitine), but accumulated long-chain acylcarnitines (linoleylcarnitine and stearoylcarnitine) (**Figure 2**). Acylcarnitines can transport fatty acids into the mitochondria, where they produce energy. Increased long-chain acylcarnitines suggesting more fatty acids entered mitochondria for energy supply by β-oxidation (Hoppel, 2003), because of decreased carbohydrates intake caused by SSd exposure. Another function of acylcarnitines is to facilitate the removal of short-and medium-chain fatty acids that accumulate in mitochondria during normal metabolic processes (Lu et al., 2016). This could explain why short-chain acylcarnitines were decreased while long-chain acylcarnitines accumulated in SSd induced liver injury. In our study, z-VAD-fmk could markedly up-regulate acetylcarnitine as well as down-regulate linoleylcarnitine and stearoylcarnitine, which indicated caspase inhibition can maintain the function of mitochondria resulted from reducing liver damage.

Histidine, with the properties of anti-oxidant, anti-inflammatory and anti-secretory (Yan et al., 2009), was observed down-regulate in SSd-treated group (**Figure 2**). Meanwhile, histidine appears to be in lower concentration in inflammation (Watanabe et al., 2008), which seems to be an inflammatory signal after SSd administration. Furthermore, markedly increased IL-1β, IL-6, and TNF-α in SSd-treated group also revealed severe inflammation (**Figures 6A,B**). The increased production of IL-β, IL-6, and TNF-α could be attributed to the activation of caspase-1 and amounts of cellular debris caused by damaged cell membranes (Laskin, 2009; Shi et al., 2015). Based on our data, caspase-1 could be induced by SSd, which resulted in the activation of IL-β and led to the damage of cell membranes. And IL-1β and/or TNFα have been shown to induce the expression of IL-6 (Starr et al., 2015), which might contribute to IL-6 level as well. So it is reasonable to consider that the increased IL-β, IL-6, and TNF-α after SSd exposure. Moreover, a decrease of LPCs and an increase of bile acids also indicated severe inflammation (Matsubara et al., 2012), as observed in SSd group. According to our result, SSd could induce severe inflammation, while caspase inhibition could significantly reverse it and lessen the liver damage.

Lysophosphatidylcholines and bile acids were screened out to be the most significant correlation with the hepatotoxicity induced by SSd and the efficacy of caspase inhibition (**Figures 3, 4**). Therefore, we focused on LPCs and biles acids in the next research. A number of changes were observed in the levels of LPCs involved in the phospholipid metabolism after SSd treatment (**Figure 2**). Previous experimental and clinical studies have showed that LPCs changes in liver diseases and hepatotoxicity models (Yang et al., 2006). Meanwhile, because LPC is a precursor of PC and PC is reported to be an important endogenous compound associated with bile acids excretion (Li and Vance, 2008), so it is reasonable to consider that the increased bile acids and the decreased LPCs. Quantitative analysis of bile acid species showed that the levels of bile acids (CA, β-MCA, TUDCA, TDCA, TCA, and TMCA) elevated in serum (**Figure 5**), which was characteristic of cholestatic liver injury caused by SSd administration. On the other hand, cholestatic liver injury often progresses to liver fibrosis, cirrhosis and eventually liver failure if left untreated (Li et al., 2017). The molecular mechanisms of bile acids induced liver injury might through mediating inflammatory response in this pathological process (Hao et al., 2017). So cholestasis might also contribute to inflammation in SSd hepatotoxicity. Disrupted phospholipid metabolism and bile acid metabolism were significant features in SSd-induced liver injury, yet caspase inhibiton could reverse both of them as to protect the liver.

More and more studies showed inflammation affected bile acids and LPCs levels in liver damage (Li et al., 2012; Tanaka et al., 2012). According to our data, bile acids and cytokines were increased and LPCs were decreased after SSd treatment. Further spearman correlation analysis was conducted to explore the underlying relationship among inflammation, bile acids, and LPCs in SSd-induced liver damage. The result demonstrated that IL-6 and TNF-α were significantly positive correlation with bile acids and were negatively related to LPCs (**Figure 6C**). It has been reported that elevated TNF-α could induce the expression of Lpcat (Tanaka et al., 2012), which could enhance the catabolism of LPCs, resulting in the decrease of LPCs. This could explain why LPCs decreased while TNF-α accumulated in SSd hepatotixicity. Meanwhile, previous studies proved that increased IL-6 and/or TNF-α might also reduce the sodium-taurocholate co-transporting polypeptide (NTCP) expression and activities (Andrejko et al., 2008; Le Vee et al., 2009; Bouezzedine et al., 2015). And the NTCP has been identified as the major bile acid uptake transporter in the basolateral membrane of hepatocytes (Ananthanarayanan et al., 1988; Meier and Stieger, 2002). The decreased NTCP transport activities affect bile acids uptake might be due to increased IL-6 and TNF-α, which could partly explain cholestasis caused by SSd treatment, as noted in our study. Therefore, the elevated IL-6 and TNF-α could contribute to decrease of LPCs and increase of bile acids. And it was reasonable to consider that IL-6 and TNF-α were positively related to bile acids and negatively to LPCs. From the above analysis, cholestasis and inflammation occurred after SSd treatment in the same time, which could accelerate each other to facilitate liver injury, while caspase inhibition could reverse both of them directly and indirectly. So caspase inhibition might provide an ameliorative effect for liver injury associated with cholestasis and inflammation in SSd hepatotoxicity.

Our study showed that caspase-1 contributed to liver injury induced by SSd, which could be effectively alleviated by z-VAD-fmk. So we deduced that caspase-1 could be a factor in the caspase inhibition reducing SSd-induced hepatotoxicity. Moreover, in consideration of the relationship between caspase-1 and inflammation, we focused on inflammation in this study. However, the caspase inhibitor, z-VAD-fmk, used in our study is a broad-spectrum inhibitor for caspase and also showed inhibitory effect on other enzymes such as lysosomal cysteine protease, cathepsin B, picornaviral 2A proteinases, and N-glycanase (Schotte et al., 1999; Deszcz et al., 2004; Misaghi et al., 2006). So the effect of other enzyme inhibition in z-VAD-fmk reduced SSd hepatotoxicity was still unknown and needed to be further studied.

## Conclusion

Caspase inhibition could reduce SSd induced-liver injury. Disrupted LPCs and bile acids were the most significant features in SSd hepatotoxicity, while caspase inhibition could restore these changes. And further analysis showed that the changes of LPCs and bile acids were likely related to the enhanced hepatic inflammatory signalings. Meanwhile, z-VAD-fmk reversed the levels of these inflammatory cytokines as well as altered LPCs and bile acids, which indicated that caspase inhibition was speculated to be key role in phospholipid and bile acid metabolism for reducing inflammation-related hepatotoxicity. Our study provided mechanistic insights into the efficacy of caspase inhibition against SSd-induced hepatotoxicity.

## Author Contributions

Q-qZ carried out most of the studies, performed the statistical analysis, and wrote the manuscript. W-qH participated in the data processing work. Y-qG and Z-dH participated in the animal experiments. WZ provided professional advices. Z-jZ and F-gX designed the study and revised the manuscript. All authors have read and approved the final version.

## Conflict of Interest Statement

The authors declare that the research was conducted in the absence of any commercial or financial relationships that could be construed as a potential conflict of interest.
