# Supplementary material for: Metabolomics Reveals the Efficacy of Caspase Inhibition for Saikosaponin D-Induced Hepatotoxicity
Source: Front Pharmacol. 2018 Jul 6;9:732. doi: 10.3389/fphar.2018.00732 (PMC6043666; doi:10.3389/fphar.2018.00732)
Supplement: Supplementary file 1 [file Presentation_1.pdf]

Supplementary Information

Metabolomics reveals the efficacy of caspase inhibition for saikosaponin d-induced hepatotoxicity

Qian-qian Zhang[1,2], Wan-qiu Huang[1,2], Yi-qiao Gao[1,2], Zhao-di Han[1,2], Wei Zhang[3], Zun-jian Zhang[1,2*], Feng-guo Xu[1,2*]

[1] Key Laboratory of Drug Quality Control and Pharmacovigilance (Ministry of Education), China Pharmaceutical University, China

[2] State Key Laboratory of Natural Medicine, China Pharmaceutical University, Nanjing, China

[3] State Key Laboratory for Quality Research in Chinese Medicines, Macau University of Science and Technology, Taipa, Macau, Macau

* Correspondence:

Zun-jian Zhang

Email: zunjianzhangcpu@hotmail.com Feng-guo Xu

Email: fengguoxu@gmail.com

Metabolomics Analysis

Sample preparation

10 μL of serum was extracted by 100 μL methanol (5 μg/mL of heptadecanoic acid in methanol), vortex for 15 min, then followed by centrifugation (14000 g ×10 min, 4 ℃) for two times, 80 μL of the supernatant was derivatized using a two-step procedure. 25 μL of MOX (10 μg/mL in pyridine) was added to the vial and shook for 90 min at 37 ℃, evaporated and followed by 120 μL MSTFA: ethyl acetate (1:4, V/V) at 37 ℃ for 120 min. The derivatized sample was for GC-MS analysis.

For LC-MS analysis, 140 μL of acetonitrile (5 μg/mL of glibenclamide in acetonitrile) was added to 20 μL of serum for protein precipitation, vortex for 5 min, the mixture was centrifuged at 16000 g for 10 min twice. The supernatant was for LC-MS analysis.

GC-MS analysis

The samples were performed on GC-MS-QP2010 Ultra (Shimadzu Co, Kyoto, Japan) equipped with a fused silica capillary column (Rtx-5MS;30 m ×0.25 mm i.d., film thickness 0.25 μm, Thames Restek, UK). The helium carrier gas flow rate was set at 1.0 mL/min. The GC oven was initially held at 70 ℃ for 3 min, and increased to 320 ℃ at a rate of 10 ℃/min, where it was held for 2 min. 1 μL of derivatized samples was injected at a split ratio of 1:50. The temperature of the injector and ion source was set at 250 ℃ and 200 ℃, respectively. The MS was operated in electron impact mode (70 eV) with a mass range of m/z 45 to 600.

LC-MS analysis

The samples were performed on Ultra Fast Liquid Chromatography (UFLC) system (Shimadzu Co, Kyoto, Japan) equipped with an electrospray ionization source operating in both positive and negative ion mode. The column held at 40 ℃ for separation was Phenomenex Kinetex C18 (100 mm ×2.1 mm, 2.6 μm) (Phenomenex, Torrance, CA, USA). The mobile phase was a mixture of (A) H_2O with 0.1% formic acid and (B) acetonitrile, with a programmed gradient as follows: linear gradient from 5% B to 95% B, 0–20 min; maintained with 95% B in 3 min, and returned to 5% B for column equilibration for 7 min. The injection volume was 5 μL and the flow rate was set at 0.4 mL/min. The scan ranges were of *m/z* 100-1000; the detector voltage was 1.7 kV; the ESI voltage was 4.5 kV in positive ion mode and -3.5 kV in negative ion mode; the ion source and heat block were maintained at 200 ℃; the nebulizing gas flow rate was 1.5 L/min; the drying gas flow was 0.1 MPa, and the ion accumulation time was 30 ms.

Data preprocessing and analysis

Each chromatogram obtained was processed by profiling solution version 1.1 (Shimadzu Co, Kyoto, Japan) for peak deconvolution and alignment. Following background-peak-filtering, 80% rule, limitation of QCs, normalization by the total ion intensity. After data preprocessing, a new data matrix containing sample names, variable names, and normalized ion intensities was imported to SIMCA-P software (version 13.0, Umetrics, Sweden) for the performance of PCA and OPLS-DA. A number of variables with

VIP value > 1.0 were identified from OPLS-DA as being responsible for the differences between treatment and control groups. Furthermore, the nonparametric Mann–Whitney U test was performed to determine the significance of each metabolite using PASW Statistics 18 (SPSS Inc., Chicago, USA).

Identification of metabolites

Compound identification in GC-MS was performed by comparing the mass fragments and intensities with NIST MS search 2.0 software (NIST, Gaithersburg, MD). Metabolites with a similarity of more than 70% were finally verified by available reference compounds. Then those metabolites were further confirmed by comparing with the standards that available in our lab. The identification of biomarkers in LC-MS followed a comprehensive strategy involving the determination of the accurate m/z, retention time, and typical MS/MS fragment and pattern. Discriminative markers were then compared with the mass-to-charge ratio (m/z), formulae and the MS/MS fragmentation of metabolites proposed by literatures and online databases, including HMDB, METLIN, Mass Bank and LIPID MAPS. Then, the putative identifications were verified by comparing the MS^n fragmentation patterns and retention time with those of authentic standard compounds that available in our lab.

Quantification of bile acids in serum

A total of 200 μL methanol and 10 μL cortisone acetate (IS, 100 μg/ mL) were added to 50 μL serum for protein precipitation, vortex for 5 min, the mixture was centrifuged at 16000 g for 10 min twice. The supernatant was for LC-MS/MS analysis. The mobile phase consisted of (A) acetonitrile and (B) 0.1% formic acid, with a programmed gradient as follows: started with 25% A for 20 min; linearly increased to 40% A in 65 min and brought back to 25% A in 5 min followed by 10 min of column equilibration. The parameters were set as follows: ESI (-), spray voltage, 3.8 kV; capillary temperature, 380 ℃; scan width for multiple-reaction monitoring (MRM), 0.1 m/z. Nitrogen was used as sheath (40 arb) and auxiliary (25 arb) gas. Argon was used as the collision gas (1.0 mTorr).

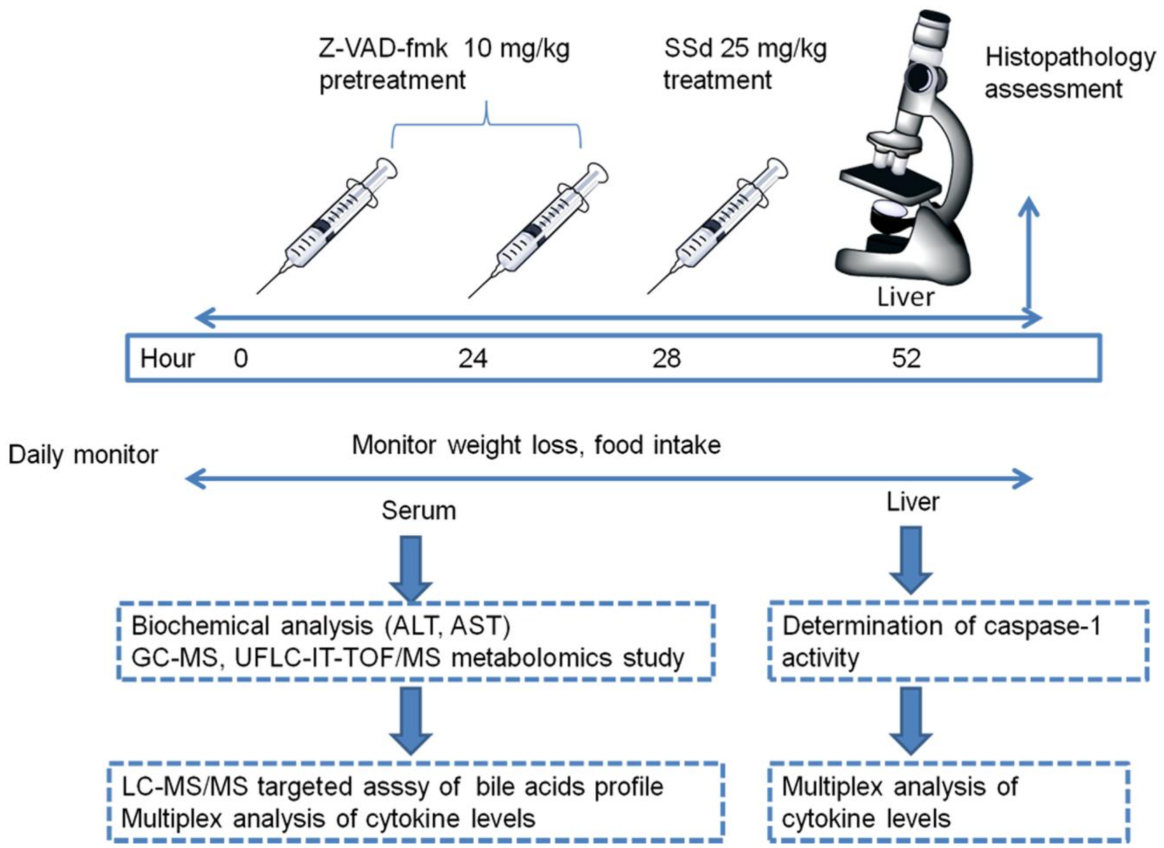

Figure S1: Experimental design. Animals either received vehicle alone, SSd alone, or SSd and z-VAD-fmk. group M and group MF were received intraperitoneal injection of 25 mg/kg of SSd at time 28 h. group MF was given z-VAD-fmk by intraperitoneal injections of 10 mg/kg at 0 h and 24 h. The weight loss and food intake of animals were monitored daily. The mice were finally sacrificed on 52 h. Serum and liver were collected for preparation and analysis.

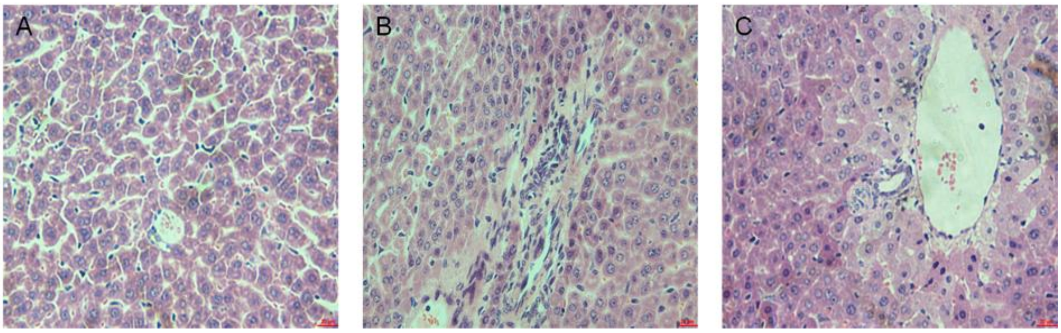

Figure S2: Representative photographs of histological examination. (A) Normal liver of group C, (B) liver of group M and (C) liver of group MF. Bar=20 μm.

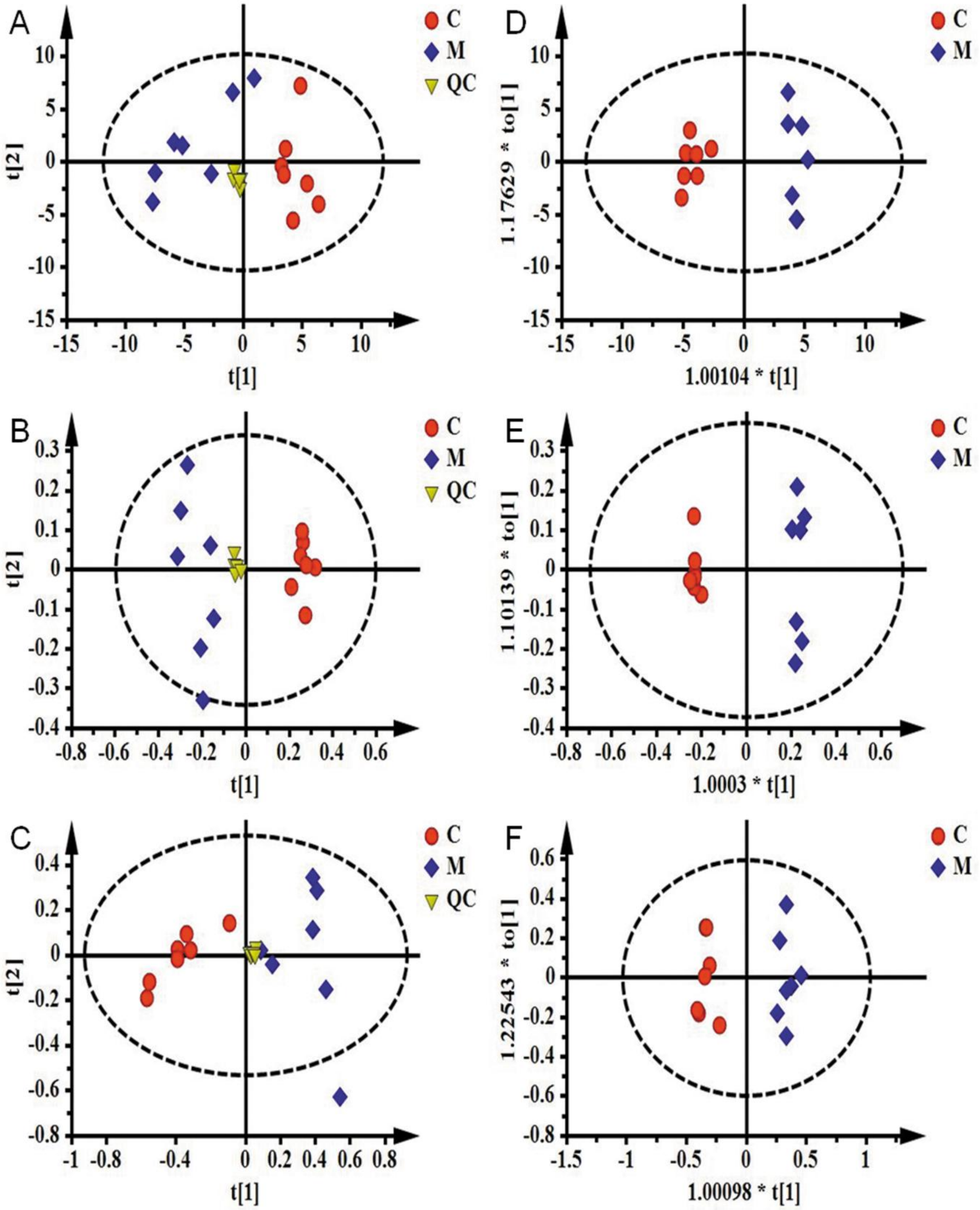

Figure S3: PCA and OPLS-DA score plots of group C and M. Score plots of PCA (A, B and C) and OPLS-DA (D, E and F) models of serum metabolite profiles after SSd (M) exposure detected by (A and D) GC-MS analysis, $R^2X=0.886$, $Q^2=0.747$; $R^2X=0.761$, $R^2Y=0.926$, $Q^2=0.867$; (B and E) LC-MS(+) analysis, $R^2X=0.578$, $Q^2=0.309$; $R^2X=0.521$, $R^2Y=0.995$, $Q^2=0.969$; (C and F) LC-MS(-) analysis, $R^2X=0.632$, $Q^2=0.345$; $R^2X=0.6$, $R^2Y=0.971$, $Q^2=0.907$. (QC, quality control).

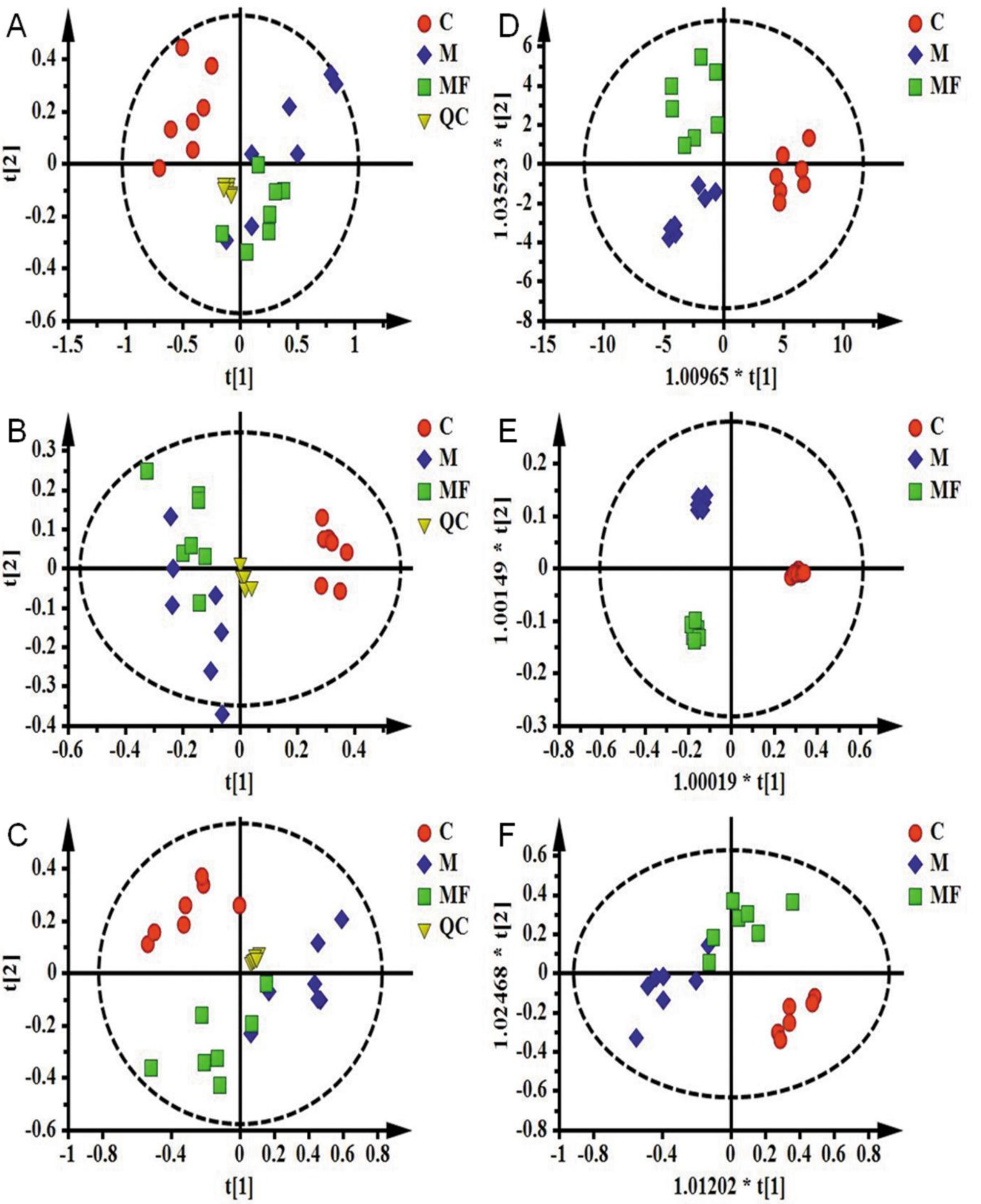

Figure S4: PCA and OPLS-DA score plots of the three groups. Score plots of PCA (A, B and C) and OPLS-DA (D, E and F) models of serum metabolite profiles after SSd (M) and z-VAD-fmk (MF) exposure detected by (A and D) GC-MS analysis, R^2X=0.891, Q^2=0.758; R^2X=0.704, R^2Y =0.686, Q^2=0.574; (B and E) LC-MS(+) analysis, R^2X=0.535, Q^2=0.326; R^2X =0.642, R^2Y=0.991, Q^2=0.757; (C and F) LC-MS(-) analysis, R^2X=0.771, Q^2=0.557; R^2X=0.592, R^2Y=0.799, Q^2=0.722. (QC, quality control).

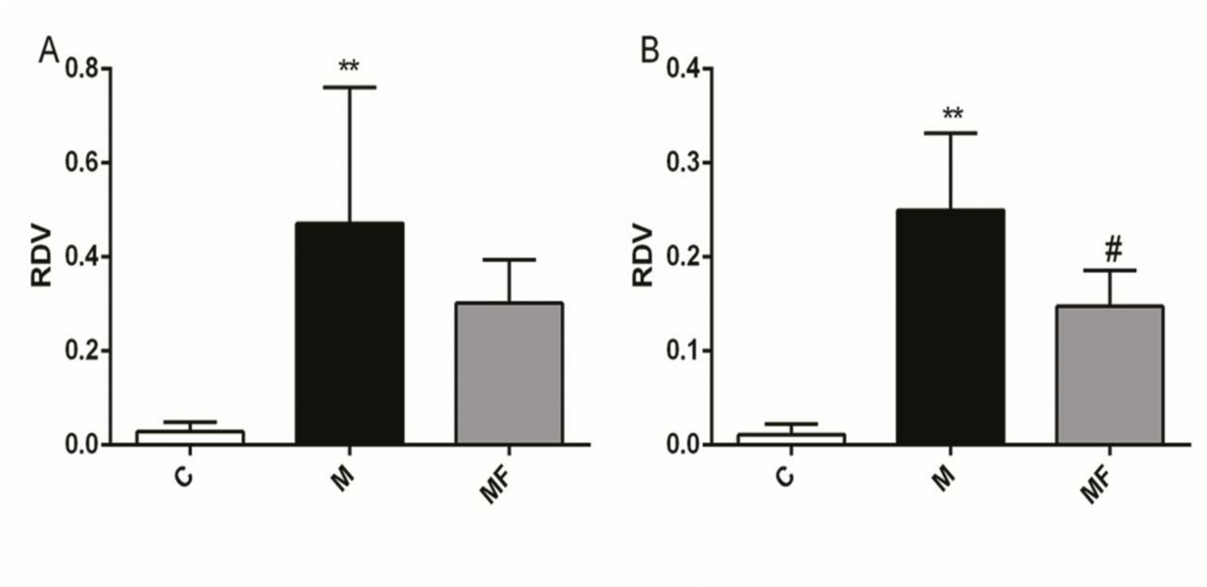

Figure S5: RDV of the three groups. RDV from (A) GC-MS and (B) LC-MS of different groups. Values are expressed as the mean ± S.D. Group M *vs*. C, *P <0.05, **P <0.01; Group MF *vs*. M, $^{\#}$P <0.05, $^{\#\#}$P <0.01.

Table S1 ALT, AST, relative body weight (RBW) and relative food intake (RFI) monitored in all groups.

Groups	ALT mean ±SD	AST mean ±SD	RBW (H52/H0%) ±SD	RFI (H52/H0%)
C	53.6 ±21.7	90.3 ±14.7	116.9 ±2.7	120.4
M	244.0 ±64.3**	1055 ±322.3**	95.1 ±2.8**	23.8
MF	121.4 ±32.5##	589.5 ±221.0##	97.5 ±2.6	31.5

Note: RBW and RFI on hour 52 were calculated relative to animals' weight and food intake at the beginning of the experiment (hour 0), respectively. Group M vs. C, *P <0.05, **P <0.01; Group MF vs. M, #P <0.05, ##P <0.01.

Table S2 List of differential metabolites in the serum of group C and M detected by GC-MS

No	Metabolite	m/z	Rt(min)	Similarity	VIP	*p*-value
	Carbohydrate and conjugates					
1	Carbohydrate 1	217.10	16.797	90	4.95365	0.002
2	Carbohydrate 2	217.10	17.365	90	6.01114	0.003
3	Carbohydrate 3	204.10	17.894	94	4.44312	0.002
4	Carbohydrate 4	205.10	17.983	91	6.33812	0.002
5	Carbohydrate 5	204.10	18.016	89	3.85493	0.013
6	Carbohydrate 6	204.10	18.771	97	3.98056	0.002
	Amino acids and derivatives					
7	Glycine	102.11	5.770	92	1.41392	0.013
8	Alanine	116.13	7.140	94	1.12178	0.035
9	Leucine	102.11	9.840	96	1.36435	0.048
10	Isoleucine	147.11	10.184	89	1.38753	0.009
11	Threonine	101.10	11.571	96	2.35533	0.004
12	Methionine	100.10	13.361	85	1.13129	0.018
13	Phenylalanine	73.06	14.749	94	1.08176	0.025
	Others				3.32777	0.002
14	Cholesterol	129.11	28.659	92	3.32777	0.002
15	Urea	147.13	9.301	92	9.47279	0.002
16	Citric acid	273.14	17.019	87	1.29856	0.048
17	Pentanedioic acid	147.11	14.058	89	1.33423	0.002

Table S3 List of differential metabolites in the serum of group C and M detected by LC-MS

No	Metabolite	m/z	Rt(min)	Adduct ions	MS/MS fragment	VIP	*p*-value
	Amino acids and derivatives						
1	Histidine	156.0420	0.571	$[M+H]^+$	140.0676	1.41024	0.003
2	Methionine	150.0576	0.667	$[M+H]^+$	/	1.32408	0.002
3	Phenylalanine	166.0864	0.797	$[M+H]^+$	120.0834	2.17863	0.004
	Carnitines						
4	Acetylcarnitine	204.1223	0.655	$[M+H]^+$	145.0473	5.42315	0.003
5	Propionylcarnitine	218.1374	0.667	$[M+H]^+$	85.0270	1.09131	0.018
6	Linoleylcarnitine	424.3426	13.312	$[M+H]^+$	189.1493	1.44322	0.013
7	Stearoylcarnitine	428.3729	15.343	$[M+H]^+$	267.2595, 369.2955	1.50689	0.002
	Glycerophospholipids						
8	LPC(14:0)	468.3078	11.811	$[M+H]^+$	450.3002, 184.0794	1.23	0.006
9	LPC(20:5)	542.3240	12.082	$[M+H]^+$	524.3098, 184.0745	2.47081	0.002
10	LPC(16:1)	494.3232	12.287	$[M+H]^+$	476.3113, 184.0706	2.3756	0.002
11	LPC(22:6)	568.3383	12.816	$[M+H]^+$	550.3271, 184.0732	1.52763	0.004
12	LPC(18:2)	564.3110	12.818	$[M+HCOO]^-$	504.2924	2.94715	0.002
13	LPC(20:4)	544.3384	12.843	$[M+H]^+$	526.3261, 184.0768	2.31527	0.002
14	LPC(22:5)	614.3217	13.111	$[M+HCOO]^-$	554.3051,	1.06415	0.002
15	LPC(20:3)	590.3245	13.326	$[M+HCOO]^-$	530.3070	1.66917	0.002
16	LPC(16:0)	540.3121	13.385	$[M+HCOO]^-$	480.2939	2.98152	0.006
17	LPC(18:1)	566.3270	13.81	$[M+HCOO]^-$	506.3063	4.08927	0.002
18	LPC(18:0)	568.3425	15.098	$[M+HCOO]^-$	508.3215	2.55761	0.003
19	LPE(20:5)	500.2762	11.905	$[M+H]^+$	482.2679, 359.2543	1.11124	0.018
20	LPE(18:2)	478.2922	12.579	$[M+H]^+$	460.2779, 337.2703	2.82207	0.002
21	LPE(20:4)	502.2925	12.603	$[M+H]^+$	484.2824, 361.2769	1.98615	0.013
22	LPE(22:6)	526.2914	12.62	$[M+H]^+$	508.2802, 385.2738	4.87399	0.002
23	LPE(22:5)	528.2990	12.633	$[M+H]^+$	510.2810	1.12961	0.006
24	LPE(16:0)	454.2920	13.096	$[M+H]^+$	436.2847	1.46036	0.006
25	LPE(18:1)	480.3437	13.943	$[M+H]^+$	462.3220	1.1997	0.002
26	LPE(18:0)	482.3235	14.642	$[M+H]^+$	464.3108	1.28074	0.018
27	Sphingosine	300.2901	12.11	$[M+H]^+$	282.2843	1.19202	0.003

28	HDoHE	343.2182	13.777	$[M-H]^-$	281.2176, 205.1181, 233.1149	1.22925	0.003
29	HETE	319.2169	13.852	$[M-H]^-$	301.2108, 179.1046, 257.2185	3.45625	0.002
	Bile acids and derivatives						
30	TMCA	514.2675	7.046	$[M-H]^-$	515.2702, 516.2687	2.3472	0.002
31	TCA	514.2669	7.966	$[M-H]^-$	515.2703, 516.2674	4.58256	0.002
	Others						
32	γ-Aminobutyric acid	104.1061	0.584	$[M+H]^+$	/	1.5784	0.018
